# Delayed Intervention for Severe Childhood Obesity in Poland: A 7-Year Gap Between Onset and Specialized Care

**DOI:** 10.3390/jcm14134726

**Published:** 2025-07-03

**Authors:** Ewa Kostrzeba, Mirosław Bik-Multanowski, Stephanie Brandt-Heunemann, Ewa Małecka-Tendera, Artur Mazur, Michael B. Ranke, Martin Wabitsch, Małgorzata Wójcik, Agnieszka Zachurzok, Katarzyna Marcinkiewicz, Anna Przestalska-Sowa, Elżbieta Petriczko

**Affiliations:** 1 Department of Pediatrics, Endocrinology, Diabetology, Metabolic Disorders and Cardiology of Developmental Age, Pomeranian Medical University, 71-252 Szczecin, Poland; 2 Department of Medical Genetics, Jagiellonian University Medical College, 31-008 Cracow, Poland; 3 University Hospital, Institute of Human Genetics, LMU, 80336 München, Germany; 4 Division of Pediatric Endocrinology and Diabetes, Department of Pediatrics and Adolescent, Medicine, University Medical Center Ulm, 89075 Ulm, Germany; 5 German Center for Child and Adolescent Health (DZKJ), Partner Site Ulm, 89075 Ulm, Germany; 6 Department of Pediatrics and Pediatric Endocrinology, Medical University of Silesia, 40-752 Katowice, Poland; 7 Department of Pediatrics, Pediatric Endocrinology and Diabetes, Institute of Medical Sciences, Medical College of Rzeszów University, 35-301 Rzeszów, Poland; 8 Children’s Hospital in Tübingen, University of Tübingen, 72076 Tübingen, Germany; 9 Department of Pediatric and Adolescent Endocrinology, Pediatric Institute, Jagiellonian University Medical College, 31-008 Cracow, Poland; 10 Department of Pediatrics, Faculty of Medical Sciences, Medical University of Silesia in Zabrze, 41-800 Zabrze, Poland

**Keywords:** childhood obesity, severe obesity, obesity prevention

## Abstract

**Background:** Childhood obesity is a growing global health concern, with an increasing prevalence of severe obesity among young children. This study aimed to determine the average age of severe obesity onset in Polish children and evaluate the time gap between diagnosis and referral for specialized care. **Methods:** This data analysis was conducted across four Polish pediatric endocrinology centers specializing in childhood obesity management (Szczecin, Cracow, Zabrze, Rzeszów) between July 2022 and November 2023. The study included 367 children and adolescents (186 boys, 181 girls) aged 0–18 years, diagnosed with severe obesity based on age-specific BMI criteria. Anthropometric measurements were performed during the patient’s inclusion into the study and based on past medical records. BMI and BMI Z-scores were calculated for all current and past measurements. **Results:** The median age of the study population at the moment of inclusion into the study was 13.7 ± 2.9 years (range: 2.2–18 years). The median BMI was 40.9 ± 5.1 kg/m^2^ (range: 30.1–65.8 kg/m^2^), and the median BMI Z-score was 2.7 ± 0.4 (range: 2.3–6.2). Out of the 367 children included, 327 (89%) had entered puberty. An analysis of past measurements revealed that 83% of children had severe obesity at their earliest recorded BMI measurement, with n median onset age of 3.2 years. The median age of referral to specialized care was 10 ± 5.0 years, reflecting a delay of almost 7 years from diagnosis to targeted medical care. **Conclusions:** This study highlights a substantial delay between the onset of severe obesity and referral for specialized care, underscoring the need for earlier intervention strategies tailored to age, sex, and developmental stage.

## 1. Introduction

Obesity is defined as an excessive or abnormal accumulation of adipose tissue that poses a risk to health. International and national statistics indicate a concerning rise in childhood obesity rates, with an increasing number of children developing obesity at a younger age. According to the World Health Organization (WHO), over 390 million children and adolescents aged 5–19 years were overweight in 2022, including 160 million living with obesity [1].

The prevalence of severe obesity varies significantly between countries, with higher rates observed in Southern Europe. An analysis of 636,933 European children revealed that the proportion of children with severe obesity ranged from 1.0% in Sweden and Moldova to 5.5% in Malta. Based on these estimates, it is projected that approximately 398,000 children aged 6–9 years in 21 European countries could be affected by severe obesity [2]. This group remains insufficiently characterized and has been the subject of relatively few scientific studies. Data from 2018 in Wales indicated that 3.1% of children aged 4–5 years met the criteria for severe obesity [3], while in 2019, 34.8% of Spanish preschool children were classified as obese, with 1.2% categorized as severely obese and 1.3% as morbidly obese [4]. In Poland, the prevalence of severe obesity among children and adolescents has not yet been thoroughly investigated.

Overweight and obesity pose serious risks for children, potentially affecting both their physical health and emotional well-being [5]. Earlier preventive and therapeutic actions create better chances of avoiding long-term health problems in adulthood [6]. Comprehensive strategies aimed at fostering healthy habits—including regular physical activity, a balanced diet, and limited screen time—have been proven effective in obesity prevention [7]. In recent years, digital tools such as mobile applications or wearable devices (e.g., bands monitoring physical activity) have emerged as promising components of pediatric obesity prevention and treatment. Despite the growing prevalence of childhood obesity, many children still lack timely support and early intervention [8]. Delays in addressing obesity risk factors may result from the absence of systematic health screenings or insufficient parental awareness and motivation [9]. Addressing these delays is crucial for preventing the progression of severe obesity and its associated complications.

Childhood obesity impacts not only physical health but also carries significant emotional and social consequences [5,10]. Psychosocial issues associated with obesity often act as both underlying and reinforcing factors, which can sustain the condition and influence treatment outcomes [10].

The aim of this study was to determine the age of onset of severe obesity in Polish children and the length of delay between onset and referral to specialist care. Understanding these time gaps is essential for improving the healthcare system and obesity treatment guidelines to ensure that children receive appropriate care at the right time.

## 2. Materials and Methods

This data analysis presents preliminary findings from a prospective, multi-center clinical investigation conducted at four Polish pediatric endocrinology centers specializing in childhood obesity management (Szczecin, Cracow, Zabrze, Rzeszow). The data were collected between 1 July 2022 and 21 November 2023 as part of the project “Prevalence of Monogenic Obesity Among Polish Children and Adolescents with Severe Obesity”, funded by the National Science Center in Poland (2021/41/B/NZ5/01676).

The final study sample included 367 children and adolescents, comprising 186 boys (51%) and 181 girls (49%), who met the following criteria:

Inclusion Criteria:

– Age range: 0–18 years.
– Body Mass Index (BMI) thresholds:
  ▪ Under 2 years: BMI > 24 kg/m^2^;
  ▪ Ages 2–6 years: BMI > 30 kg/m^2^;
  ▪ Ages 6–14 years: BMI > 35 kg/m^2^;
  ▪ Over 14 years: BMI > 40 kg/m^2^;
  ▪ A documented history of severe obesity.
– The presence of hyperphagia and food-seeking behavior, assessed using validated questionnaires appropriate for age (CEBQ, TFEQ, or HQ-CT).
– Written informed consent from the parent/guardian or the patient (if over 13 years old).

Exclusion Criteria:

– The absence of signed informed consent
– Obesity caused by secondary factors, including previously diagnosed genetic syndromes or endocrine disorders associated with obesity, medication use that is known to contribute to weight gain, or other identified secondary causes of obesity.

Data were obtained during a single visit to the medical center. Each patient underwent a physical examination, including anthropometric measurements: body weight was measured to the nearest 0.1 kg on a certified medical scale, and height was measured to the nearest 0.1 cm using a Harpenden stadiometer. Additional historical anthropometric data (from hospitalizations, check-ups, or vaccination qualifications) were collected from medical records when available. Based on these data, BMI and BMI Z-scores were calculated for both current and past measurements. BMI was calculated using the following formula:

(1) $$BMI=\frac{weight[kg]}{height[m^{2}]}$$

BMI Z-scores were obtained for children aged 2–18 using the Pediatric Z-Score Calculator [11]. The BMI Z-score is a measure that indicates how many standard deviations (SDs) a child’s Body Mass Index (BMI) is derived from compared to the average BMI for children of the same age and sex in a reference population. It allows for the age- and sex-specific assessment of nutritional status. Severe obesity was defined as a BMI Z-score > 2 SD in historical records [12]. The full study methodology is detailed elsewhere [13].

Categorical variables were analyzed using Fisher’s exact test. Due to deviations from normality, differences in continuous data were assessed with the nonparametric Wilcoxon rank sum test. A *p*-value < 0.05 was considered statistically significant. All calculations and visualizations were performed using the R statistical environment (R Foundation for Statistical Computing, Vienna, Austria, version 4.3.2, 2023).

## 3. Results

The study included 367 children with severe obesity (181 girls, 186 boys), with a median age of 13.7 years (14 for girls, 13.6 for boys; *p* = 0.006). Most participants (89%) had entered puberty (Tanner stage ≥ II), with a median age of 14 years, while the prepubertal group (11%) had a median age of 9.1 years (*p* < 0.001). Boys predominated among prepubertal children (80%), while sex distribution was more balanced in the pubertal group.

The median BMI was 40.9 kg/m^2^ (41.4 for girls, 40.3 for boys) and was higher in pubertal children (41.3 kg/m^2^) compared to prepubertal children (38.3 kg/m^2^; *p* < 0.001). The overall median BMI Z-score was 2.65 and was higher in boys (2.7) than girls (2.6) and in prepubertal (2.8) compared to pubertal children (2.3; *p* < 0.001). Details are presented in Table 1.

Out of 367 children included, 256 provided at least one past medical measurement, with the first exam taken at a median age of 3.2 years. The median BMI at that time was 22.2 kg/m^2^, with no significant differences by sex (*p* = 0.7) or pubertal status at inclusion (*p* = 0.3). The median BMI Z-score was 2.63, and 83% of the children met the criteria for severe obesity (BMI Z-score > 2.0) at their first measurement.

Among the 169 children with at least two past measurements, the second was taken at a median age of 6.1 years. The median BMI increased to 26.1 kg/m^2^, again without significant sex (*p* = 0.2) or pubertal status differences (*p* = 0.2). The median BMI Z-score was 2.64, significantly higher in boys (2.79) than girls (2.52; *p* < 0.001). Children who were prepubertal at inclusion showed higher BMI Z-scores at the second measurement compared to pubertal peers (3.45 vs. 2.61; *p* < 0.001). At this time, 86% met the criteria for severe obesity.

In total, 95 children provided three past medical measurements, with the third taken at a median age of 9.4 years. The median BMI was 33.1 kg/m^2^, without significant differences in sex (*p* = 0.4) or pubertal status (*p* = 0.2). The median BMI Z-score was 2.61 and was significantly higher in boys (2.71) than girls (2.52; *p* = 0.001). Prepubertal children had higher BMI Z-scores at the third measurement compared to their pubertal peers (3.04 vs. 2.58; *p* < 0.001). At this time, 95% met the criteria for severe obesity.

A total of 12 children delivered their past four measurements. The fourth measurement was taken at a median age of 10.8 years, with a median BMI of 37.2 kg/m^2^ and a BMI Z-score of 2.62, showing no sex differences (*p* = 0.6).

All children started multi-specialty care at a median age of 10.2 years. Prepubertal children began receiving specialized care significantly earlier than pubertal children (5.5 vs. 11.5 years; *p* < 0.001). The BMI increased steadily with age, while the BMI Z-scores remained elevated but relatively stable, indicating consistent obesity levels across time.

The details are presented in Table 2.

## 4. Discussion

### 4.1. BMI Trends by Sex, Age, and Developmental Stage

The results of this study highlight the complexities of BMI trends and obesity prevalence among children, reflecting an interaction between age, sex, and developmental stages. Analyzing trends in the increased prevalence of severe obesity by sex and age can be crucial for designing intervention programs tailored toward targeting the hormonal and psychological changes occurring at specific developmental ages.

BMI values for children aged 0 to 5 typically follow a predictable growth pattern that is relatively similar across sexes. In infancy, BMI values are higher due to rapid weight gain relative to length, peaking at around 6–12 months. The body fat percentage of a newborn equals around 15%, while at the end of the first year of life, this value increases to 25%. This is called the first critical period of fat tissue development. A significant increase during this period greatly raises the risk of developing obesity in the future, which is known as “metabolic programming” [14].

BMI gradually declines from 12 months of age until it reaches a low point between ages 4 and 6. This decline happens as children become more active and grow taller. Five-year-olds are characterized by a “hunger” for movement; they draw social models for physical activity from significant individuals who can shape their behaviors in this area. After the age of 6, BMI begins to rise again, which is known as the “adiposity rebound.” An early adiposity rebound (before 5.5 years) has been widely considered an indicator of potential obesity in later life [15].

Growth and weight patterns may vary individually due to genetic and environmental factors. The results of our study indicated a rapid increase in obesity rates when obesity onset occurred at a younger age, which might potentially be exacerbated by the excessive weight gain during the first critical period of fat tissue development as well as adiposity rebound.

It is striking that the average BMI of children in the prepubertal period, typically up until the age of 8, was equal to 38.9 kg/m^2^, which was only slightly lower than the average BMI of children in the pubertal period at 41.6 kg/m^2^. This underscores the scale of the problem of obesity among young children, which is connected with a higher risk of developing cardiometabolic complications [16]. Excess weight gain in prepubertal children can also lead to an acceleration in linear growth velocity and premature pubertal development in girls, while it may delay puberty in boys [17]. In subsequent years, growth velocity slows down, and children tend to reach a similar final height.

Boys see significant weight gain around ages 7–8, which is connected with an early increase in muscle mass and bone density. This initial weight increase serves as a foundation for their later growth spurt in mid-adolescence, when rising testosterone levels further promote muscle and bone development, preparing for the greater physical demands that come with later childhood [18]. For both sexes, growth hormones and thyroid hormones play significant roles in body development, but sex hormones (testosterone in boys and estrogen in girls) affect the timing and distribution of body fat differently. Increased estrogen levels at the age of 10–14 in girls lead to greater body fat distribution around the hips, thighs, and chest in preparation for reproductive maturity.

Increased screen time and engagement with social media are common during adolescence. Prolonged screen times correlate with an individual’s lifestyle, leading to reduced calorie expenditure, increased weight gain, anxiety, and depression [19,20]. This age group tends to consume more high-calorie, low-nutrient foods, which are easily accessible and marketed extensively on social media. Additionally, exposure to idealized body images on social media can affect self-esteem and mental health, which may contribute to emotional eating and irregular eating patterns [21]. The excessive use of screens imitating blue light affects sleep patterns, which is associated with metabolic changes, such as decreased insulin sensitivity, increased appetite-regulating hormones (e.g., ghrelin), poor dietary choices, and decreased physical activity [22]. Furthermore, the children in this study cohort experienced the lockdown imposed due to the COVID-19 pandemic. A systematic review (PROSPERO: CRD42024589208) of English-language studies published up to 1 October 2024, investigated physical activity levels before, during, and after the pandemic in individuals aged 6 to 18 years, with a particular focus on BMI and weight status in relation to age and sex. The review included 26 studies covering a total of 138,737 children and adolescents. The findings showed a reduction in physical activity, an increase in sedentary behaviors during and after the lockdown, and a rise in BMI and the prevalence of overweight and obesity, especially among boys and children aged 8 to 11 years [23].

### 4.2. Diagnostic Delay and the Need for Early Intervention

A troubling finding is that children reach multi-specialty care with an average delay of 4 years, by which time 83% have already met the criteria for severe obesity in initial past measurements. This raises the question of what causes such a large time gap and how to improve access to early multi-specialty care interventions. A survey conducted in Louisiana found that although 88% of pediatricians screen for obesity, only 7% follow the recommended guidelines for referring patients to pediatric weight management support [24].

Certainly, there is room for initiatives made by family doctors and specialists alike, who could monitor and act on early signs by conducting regular measurements and referring overweight children to specialized metabolic care. However, many parents do not bring their children for routine health check-ups and avoid mandatory childhood vaccinations, which limits the opportunities for family doctors to thoroughly examine children outside of visits related to infections. Moreover, in Poland, the treatment of children with obesity is managed by pediatric endocrinology specialists. Unfortunately, the average waiting time for hospitalization in a Pediatric Endocrinology Clinic can be as long as a year, while the first appointment at a Metabolic Diseases Outpatient Clinic may take up to two years. During this time, body weight typically increases significantly, reducing the effectiveness of medical, dietary, and psychological interventions. This issue has been recognized by the Polish government. Currently, a National Program for the Prevention and Treatment of Obesity is being developed in Poland. As part of this initiative, family doctors will receive the necessary resources and tools needed to quickly respond to progressive weight gain in children.

According to the 2022–2024 findings presented in the sixth round of the WHO European Childhood Obesity Surveillance Initiative (COSI), the highest prevalence of obesity among children aged 7–9 years was observed in Cyprus, where 42% of children were affected. Poland also ranks high, with approximately 33% of children in this age group classified as being overweight or obese. In contrast, the lowest prevalences of obesity in European countries were reported in the Czech Republic and Denmark, at 21% and 20%, respectively [25]. The Czech Republic has introduced school food regulations banning unhealthy products, along with national guidelines to improve meal quality. Health education and physical activity are promoted through school programs and public campaigns. The government also monitors compliance in school canteens. The Czech Republic also utilizes digital tools to combat childhood obesity [26]. The pilot program Buď Fit 24 targets overweight children aged 6–11, providing them with free fitness trackers and a mobile app to monitor physical activity and calorie intake. This innovative program, in cooperation with healthcare professionals, aims to reduce BMI by 5% within a year while promoting healthy lifestyles and family support [27]. Intervening simultaneously in key areas seems to contribute to success in maintaining low childhood obesity rates.

When it comes to strategies implemented by Denmark, there is a strong emphasis on creating nutrition-friendly schools and integrating nutrition education into both school and community programs. These strategies also focus on conducting research on child health in order to develop broad, system-level interventions supported by scientific evidence. Many of these programs actively involve local communities—for example, the Generation Healthy Kids study, which maps local risk factors and identifies opportunities for obesity prevention [28]. Compared to the more structured, multi-sectoral approaches in Denmark and the targeted regulatory and digital strategies in Czechia, Poland’s childhood obesity prevention efforts lack cohesive national coordination, long-term evaluation, and integration across education, healthcare, and community systems.

To date, most strategies to prevent overweight and obesity in Poland have focused on school-aged children, encouraging individuals to adopt healthier habits, like increasing physical activity and improving diet by reducing excess calories [29]. One of them was the intervention program called “Courageous Eight”, targeted at 8- and 9-year-old children with excess body weight living in Szczecin, Poland. It was conducted in 2016–2018 in two phases: screening in elementary schools and an intervention at the outpatient clinic of the Pomeranian Medical University hospital, involving 515 children. The one-year multi-specialty intervention program turned out to be effective, as participants stopped gaining weight, so their anthropometric indicators such as BMI, BMI centile, and BMI z-score decreased. Despite improvements in anthropometric measures, many children continued to exhibit metabolic disturbances, including abnormal glucose, insulin, and lipid levels [30,31]. Preliminary results from studies analyzing the occurrence of cardiovascular risk factors in our study population showed that among the 140 children with severe obesity, 89% had elevated blood pressure, 84% had lipid abnormalities, 19% had hyperglycemia, and only 9% were free of metabolic complications when tested. The main conclusion from this analysis was that the most important factor determining the presence of obesity complications, and thus the total metabolic risk was an earlier age of obesity onset (<5 years) [16]. These results suggest that implementing more programs for younger children, including additional general developmental physical activity sessions, could be beneficial. In our study, 83% of children met the criteria for severe obesity by the age of five. Early interventions may help prevent metabolic disturbances before they become persistent, increasing the chances of long-term weight normalization and better health outcomes.

It is possible that the future of obesity treatment lies in improving care through the integration of digital health tools, such as telemedicine consultations, digital growth charts, and mobile applications, which may facilitate the earlier identification of children at risk and support more timely interventions. These technologies may be particularly relevant for adolescents, an underserved age group for which preventive programs are not typically designed. Given their strong engagement with digital platforms, adolescents increasingly derive motivation from social media and peer groups, spending a significant amount of time in front of screens. This behavioral pattern creates both challenges and opportunities for intervention. The first promising results of integrating mobile health technology with in-person visits have already been described in the literature. Evira includes a digit-less body scale for home-weighing, a mobile app, and a web-based clinical interface, facilitating communication between families and healthcare providers while offering continuous visual feedback on treatment progress. A total of 107 children with obesity aged 4.0–17.9 years received digi-physical treatment with Evira, while 321 children followed standard care. After three years, children in the digi-physical treatment group achieved more than twice the average relative weight loss compared to those receiving standard care (−0.29 vs. −0.12 BMI Z-score units), with particularly significant improvements in adolescents (−0.38 vs. 0.02 BMI Z-score units). Overall, the digi-physical approach demonstrated a greater treatment effect and a higher obesity remission rate over three years compared to traditional treatment methods [32]. Our findings suggest that the main difficulties adolescents encounter are a lack of a sense of belonging and feelings of being neglected. Evira offers a communication platform through written messages and effectively detects patients who require additional support. Its communication approach, based on motivational interviewing, prioritizes empowerment rather than education, which may explain its effectiveness, particularly among adolescents. Integrating digital tools into early intervention models could be a great opportunity to enhance the effectiveness and scalability of pediatric obesity treatment.

A notable limitation of the study was the limited number of historical medical measures available. In several cases, parents did not provide complete medical documentation, which restricted the comprehensiveness of the data. Additionally, the available measurements were obtained using different medical scales and by various healthcare professionals, which might have introduced inconsistencies in the data collection process. To mitigate these limitations, efforts were made to verify the accuracy and consistency of the reported data by cross-referencing with available electronic health records where possible. Furthermore, standardized measurement procedures were applied during the study period to ensure consistency in data collection moving forward. The other limitation of this study was the reliance on BMI as inclusion criteria. Although not ideal, it was chosen due to its widespread application as the screening indicator of obesity. In order to ensure standardization and allow for comparison across ages and sexes, BMI Z-score values were calculated. Unlike raw BMI values, which can be difficult to interpret in growing children, BMI Z-scores allow for meaningful comparisons and changes to be tracked over time. The strengths of this study include its focus on a poorly characterized but clinically significant subgroup of children with severe obesity, using a multi-center cohort from four regional reference centers in Poland. What is more, it presents longitudinal data from early childhood to adolescence and highlights the association between an early age at obesity onset and the presence of metabolic complications.

## 5. Conclusions

Our findings reinforce the urgency of implementing comprehensive, age-specific, and long-term strategies to prevent and manage childhood obesity. Early and accessible interventions, combined with innovative digital tools and family-centered care, could significantly improve health outcomes and reduce the burden of obesity-related complications in children and adolescents.

## Figures and Tables

**Table 1:** Selected characteristics and derived parameters of the study population on the day of their inclusion into the study (severe obesity diagnosis).

Whole Study Population			Sex			Pubertal Period		
Characteristic	Available Data	Overall, *n* = 367 ^1^	Girls, *n* = 181 ^1^	Boys, *n* = 186 ^1^	*p*-Value ^2^	Puberty, *n* = 327 ^1^	Prepuberty, *n* = 40 ^1^	*p*-Value ^2^
Age (Years)	367	2.24–18.01 (13.74/2.89)	2.76–18.01 (13.99/2.58)	2.24–17.91 (13.60/3.10)	0.006	4.17–18.01 (14.04/2.27)	2.24–15.84 (9.14/3.01)	<0.001
Sex	367							<0.001
Girls		181 (49%)				173 (53%)	8 (20%)	
Boys		186 (51%)				154 (47%)	32 (80%)	
Pubertal Period	367				<0.001			
Puberty		327 (89%)	173 (96%)	154 (83%)				
Prepuberty		40 (11%)	8 (4.4%)	32 (17%)				
BMI (kg/m ^2^)	367	30.05–65.77 (40.9/5.10)	30.39–57.66 (41.35/4.78)	30.05–65.77 (40.26/5.40)	0.12	30.39–65.77 (41.28/4.89)	30.05–62.08 (38.28/6.13)	<0.001
BMI Z-score	367	2.27–6.16 (2.65/0.42)	2.27–4.89 (2.55/0.24)	2.48–6.16 (2.74/0.50)	<0.001	2.27–6.01 (2.63/0.29)	2.53–6.16 (2.83/0.83)	<0.001

^1^ Range (median/SD), ^2^ Wilcoxon rank sum test.

**Table 2:** The selected characteristics and derived parameters of the study population for past measurements.

Characteristic	Available Data	Overall, *n* = 367 ^1^	Girls, *n* = 181 ^1^	Boys, *n* = 186 ^1^	*p*-Value ^2^	Puberty, *n* = 327 ^1^	Prepuberty, *n* = 40 ^1^	*p*-Value ^2^
**1st measurement from the past**								
Age (years)	256	0.16–17.38 (3.22/4.43)	0.21–17.38 (3.62/4.49)	0.16–16.07 (3.06/4.39)	0.8	0.16–17.38 (3.79/4.52)	0.42–11.43 (2.01/3.08)	0.011
BMI (kg/m^2^)	256	10.18–68.49 (22.21/9.27)	10.18–68.49 (23.21/9.37)	12.05–57.77 (21.74/9.20)	0.7	10.18–68.49 (22.05/9.54)	16.46–41.50 (24.44/6.76)	0.3
BMI Z-score	168	0.46–5.67 (2.63/0.79)	0.46–4.49 (2.59/0.71)	0.61–5.67 (2.65/0.86)	0.5	0.46–5.67 (2.62/0.77)	0.61–4.49 (2.81/1.04)	0.2
Diagnosis of severe obesity	168				0.8			>0.9
No		29 (17%)	15 (18%)	14 (16%)		27 (18%)	2 (14%)	
Yes		139 (83%)	68 (82%)	71 (84%)		127 (82%)	12 (86%)	
**2nd measurement from the past**								
Age (years)	169	1.21–17.03 (6.11/3.67)	1.21–15.41 (6.23/3.52)	2.06–17.03 (6.03/3.85)	>0.9	1.21–17.03 (6.29/3.74)	2.06–10.82 (4.49/2.24)	0.023
BMI (kg/m^2^)	169	13.97–53.22 (26.13/8.35)	13.97–45.91 (25.03/7.82)	16.63–53.22 (26.3/8.83)	0.2	13.97–53.22 (25.65/8.53)	20.73–39.30 (28.77/6.43)	0.2
BMI Z-score	168	0.35–6.59 (2.64/0.91)	0.35–4.12 (2.52/0.64)	0.76–6.59 (2.79/1.07)	<0.001	0.35–5.38 (2.61/0.78)	2.53–6.59 (3.45/1.23)	<0.001
Diagnosis of severe obesity	168				0.3			0.14
No		24 (14%)	15 (17%)	9 (11%)		24 (16%)	0 (0%)	
Yes		144 (86%)	72 (83%)	72 (89%)		127 (84%)	17 (100%)	
**3rd measurement from the past**								
Age (years)	95	4.06–16.87 (9.38/3.19)	4.06–16.61 (10.05/3.01)	4.86–16.87 (9.31/3.38)	0.3	4.06–16.87 (9.80/3.19)	5.08–9.94 (6.80/1.79)	0.022
BMI (kg/m^2^)	95	18.67–52.22 (33.09/7.99)	19.14–47.16 (33.16/6.85)	18.67–52.22 (33.09/9.05)	0.4	18.67–52.22 (33.07/7.90)	25.54–50.21 (34.63/8.74)	0.2
BMI Z-score	94	1.80–4.19 (2.61/0.39)	1.85–3.12 (2.52/0.30)	1.80–4.19 (2.71/0.43)	0.001	1.80–3.81 (2.58/0.34)	2.69–4.19 (3.04/0.55)	<0.001
Diagnosis of severe obesity	94				0.4			>0.9
No		5 (5.3%)	4 (8.2%)	1 (2.2%)		5 (5.7%)	0 (0%)	
Yes		89 (95%)	45 (92%)	44 (98%)		82 (94%)	7 (100%)	
**4th measurement from the past**								
Age (years)	12	6.39–17.91 (10.75/ 3.17)	6.39–17.91 (10.15/3.62)	8.63–15.82 (11.35/2.80)	0.8	6.39–17.91 (10.75/3.17)		
BMI (kg/m^2^)	12	26.52–42.34 (37.22/6.25)	26.52–42.30 (36.57/6.62)	27.34–42.34 (39.68/6.34)	0.6	26.52–42.34 (37.22/6.25)		
BMI Z-score	12	2.13–2.94 (2.62/0.25)	2.13–2.94 (2.50/0.29)	2.26–2.74 (2.66/0.20)	0.6	2.13–2.94 (2.62/0.25)		
Diagnosis of severe obesity	94				0.4			>0.9
No		5 (5.3%)	4 (8.2%)	1 (2.2%)		5 (5.7%)	0 (0%)	
Yes		89 (95%)	45 (92%)	44 (98%)		82 (94%)	7 (100%)	
**Time to diagnosis**	367	2.01–18.01 (10.02/4.96)	2.01–18.01 (10.15/4.86)	2.01–17.91 (9.57/5.06)	0.4	2.01–18.01 (11.53/4.96)	2.03–15.84 (5.48/3.39)	<0.001

^1^ Range (median/SD), ^2^ Wilcoxon rank sum test.

## Data Availability

The raw data supporting the conclusions of this article will be made available by the authors upon request.

## Abbreviations

The following abbreviations are used in this manuscript:

BMI	Body Mass Index
BMI Z-score	Body Mass Index Z-score
